# Risk factors for childhood obesity: shift of the entire BMI distribution vs. shift of the upper tail only in a cross sectional study

**DOI:** 10.1186/1471-2458-8-115

**Published:** 2008-04-10

**Authors:** André M Toschke, Rüdiger von Kries, Andreas Beyerlein, Simon Rückinger

**Affiliations:** 1 King's College London, Division of Health and Social Care Research, Department of Public Health Sciences, 7th Floor Capital House, 42 Weston St, London, SE1 3QD, UK; 2 Ludwig-Maximilians-University of Munich, Division of Pediatric Epidemiology at the Institute of Social Pediatrics and Adolescent Medicine, Munich, Germany

## Abstract

**Background:**

Previous studies reported an increase of upper body mass index (BMI) quantiles for formula fed infants compared to breastfed infants, while corresponding mean differences were low. The aim of this study was to assess the impact of known risk factors for childhood obesity on the BMI distribution.

**Methods:**

Data on 4,884 children were obtained at obligatory school entry health examinations in Bavaria (Germany). Exposure variables were formula feeding, maternal smoking in pregnancy, excessive TV-watching, low meal frequency, poor parental education, maternal overweight and high infant weight gain. Cumulative BMI distributions and Tukey mean-difference plots were used to assess possible shifts of BMI distributions by exposure.

**Results:**

Maternal overweight and high infant weight gain shifted the entire BMI-distribution with an accentuation on upper quantiles to higher BMI values. In contrast, parental education, formula feeding, high TV consumption, low meal frequency and maternal smoking in pregnancy resulted in a shift of upper quantiles only.

**Conclusion:**

The single shifts among upper parts of the BMI distribution might be due to effect modification of the corresponding exposures by another environmental exposure or genetic predisposition. Affected individuals might represent a susceptible subpopulation of the exposed.

## Background

For most known environmental risk factors only a proportion of those exposed will be affected by subsequent categorical outcomes such as death or disease. This may be due to the dose of exposure or interaction with individual genetic disposition or interactions with other known or unknown risk factors. Although childhood obesity is a categorical outcome by definition, it is usually defined by application of arbitrary cut-off values to continuous measures such as BMI, skin fold thickness or percentage of fat in the body composition [1-3]. An increase of the obesity prevalence related to a specific risk factor may arise e.g. from a shift in the entire distribution of the BMI or a shift of the upper tail only [4].

If all exposed individuals in a population under study were exposed to the same dose of a risk factor for obesity and this risk factor also affects all these individuals, a shift of the entire BMI distribution among the exposed would be expected. A risk factor for obesity affecting a proportion of those exposed only, because an interaction with another risk factor is required, would account for a single shift of the upper tail of the BMI distribution. A genetic predisposition of an exposed subpopulation or an interaction with other known or unknown risk factors might account for this interaction.

The BMI distribution is commonly used to define overweight and obesity. While in the US the 85^th ^and 95^th ^percentile are applied to define overweight and obesity in children[5] the European recommendations proposed the use of the 90^th ^and 97^th ^percentiles respectively[2,3]. Interestingly in children and young adults the epidemic does not appear to affect the entire population equally. While BMI values within lower quartiles remain almost unchanged, clear BMI increases are observed in the top quartile[4,6-10].

A number of known risk factors for childhood obesity have been identified such as excessive TV-watching, poor parental education, maternal overweight, high weight gain in the first 2 years of life, formula feeding, maternal smoking in pregnancy and low meal frequency. Their impact on the BMI distribution, however, has not been studied yet. We aimed to study the shift or increased skewness, respectively, of the BMI distribution for each of those risk factors.

## Methods

### Study population and data sources

During the year before school entry all children in Bavaria have to attend at the mandatory school entry health examination in local public health offices. The purpose of this compulsory examination is to assess deficits which might influence school performance (i.e. impaired visual faculty) but can easily be corrected (i.e. prescription of glasses). Most of the children are at age 5 and 6 years when examined. Parents of 8,741 children were invited to participate in a voluntary self-completion questionnaire study as part of their child's obligatory school entry examination in six Bavarian communities (Germany) from September 2001 to August 2002. Questionnaires were mailed together with the invitations for the school entry health examination. About 80 percent (n = 7,026) completed questionnaires were returned. Data on a number of sociodemographic and potential risk factors for childhood obesity were linked with children's stature and weight measures. The study was approved by the Bavarian State Office for Data Protection and the local ethics committee.

The analysis was confined to 4,884 children with full information on age, anthropometric measures, maternal BMI, maternal smoking in pregnancy, TV watching, weight gain in the first 24 months, low meal frequency, breastfeeding and parental education.

### Measures

Stature and weight were measured in light clothing and without shoes by trained nurses of local public health offices. Stadiometers and balances were periodically calibrated by respective gauging offices.

A number of risk factors with previously reported associations with childhood obesity were considered as exposures. All variables were dichotomized to compare BMI distributions of exposed and non-exposed individuals. Low educational level of parents was assumed when neither father nor mother achieved O-level [11]. Mothers with a body mass index of at least 25 kg/m^2 ^were classified as overweight [12,13]. High weight gain in the first two years of life [14] was assumed if the difference between reported birth weight and weight at well baby check-up at 24 months was more than 10 kg [15]. Low meal frequency was assumed for children consuming less than 5 meals a day [16]. Children watching television for more than 1 hour a day were classified as having high TV consumption [17-19]. Furthermore the variables smoking in pregnancy and breastfeeding were included in the analysis as binary variables [20-26].

### Statistical analysis

Tukey mean-difference plots visualize a comparison between quantiles of two distributions. In our case the distributions of BMI for a population of exposed and a population of non-exposed children were compared. While the horizontal axis shows the mean of the two quantiles of both distributions, the vertical axis shows the difference of the same two quantiles. For example, let the 10^th ^percentile of the exposed population be 21 and the 10^th ^percentile of the non-exposed population be 20. Then the corresponding mean is 20.5 (horizontal axis) with a difference of 1 (vertical axis) and the point (20.5, 1) will be plotted.

If two distributions are exactly the same, then all quantiles are equal and subsequently all differences between quantiles are zero, resulting in a mean-difference plot which represents a horizontal line at zero. If two distributions only differ in their central tendency the mean-difference plot represents a horizontal line at the respective mean difference between those two distributions. If distributions only differ in the upper quantiles, the mean-difference plot takes an increasing form, starting from zero.

BMI cumulative distributions by exposure category and mean-difference plots as well as all calculations were carried out using the statistical software package R version 2.6.1 [27].

## Results

**Figure 1:**
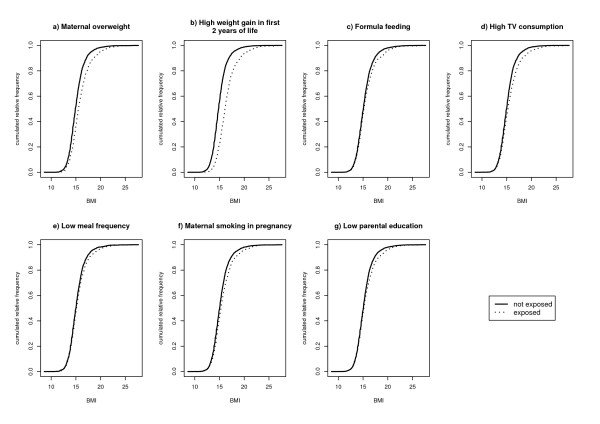
Cumulative BMI distributions of exposed and non-exposed individuals to certain risk factors.

Figure 1 shows the cumulative BMI distribution of exposed and non-exposed children by different risk factors. For maternal overweight and high weight gain in the first two years of life a shift of the entire BMI-distribution with an accentuation on upper quantiles could be observed (fig. 1a and 1b). In contrast, the risk factors parental education, formula feeding, high TV watching, low meal frequency and maternal smoking in pregnancy suggest a shift of upper qantiles only while there is almost no shift of the lower end of the distribution (fig. 1c–g). Corresponding BMI values at the 25^th^, 50^th ^(median), 75^th^, and 95^th ^percentile for children exposed/not exposed to specific risk factors are presented in Table 1.

**Table 1:** BMI values in kg/m^2 ^at the 25^th^, 50^th^, 75^th ^and 95^th ^percentile for children exposed/not exposed to respective risk factor

Risk factor	Exposed				Not Exposed			
		
	25^th^	50^th^	75^th^	95^th^	25^th^	50^th^	75^th^	95^th^
Maternal overweight	14.6	15.6	17.0	22.7	14.1	14.9	16.0	18.6
High weight gain in first 2 years of life	15.1	16.1	17.3	20.7	14.0	14.8	15.7	17.8
Formula feeding	14.3	15.1	16.4	19.7	14.1	15.0	16.1	18.3
High TV consumption	14.3	15.3	16.6	19.7	14.1	15.0	15.9	18.1
Low meal frequency	14.2	15.1	16.3	19.2	14.1	15.0	16.0	18.1
Maternal smoking in pregnancy	14.4	15.3	16.6	19.5	14.1	15.0	16.0	18.5
Low parental education	14.2	15.2	16.4	19.5	14.1	15.0	16.1	18.3

**Figure 2:**
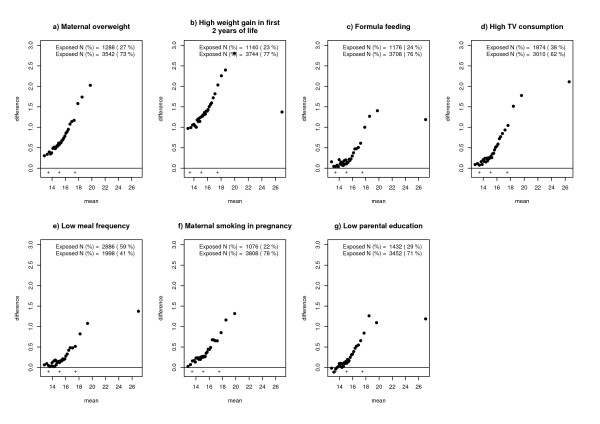
Tukey mean difference plots of BMI distributions of exposed and non-exposed individuals to certain risk factors.

These associations are underlined by mean-difference plots shown in figures 2a–g. A shift of BMI especially in the upper tail of the distribution could be observed for all risk factors and is represented by an increasing difference among higher quantiles. Again, a shift of the entire BMI distribution could be observed for maternal overweight and high infant weight gain, however, with an increasing tendency among upper quantiles. A single shift of the upper tail of the BMI distribution could be observed for poor parental education, watching TV, formula feeding, low meal frequency and smoking in pregnancy. For the latter exposures no differences could be observed among lower percentiles accompanied by large differences among higher quantiles.

**Figure 3:**
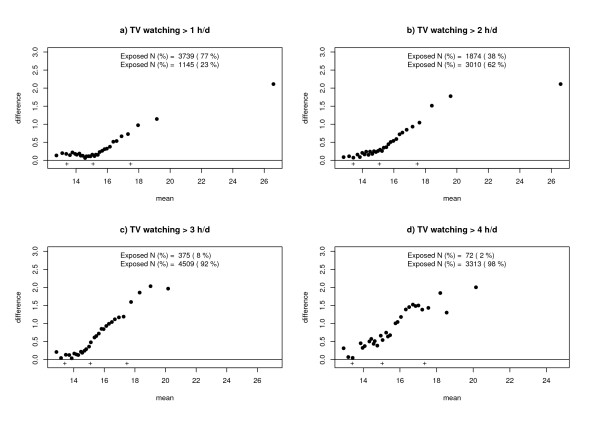
Tukey mean difference plots of BMI distributions of exposed and non-exposed individuals to watching TV by different cut-points.

To assess possible dose response effects on parts of the BMI distribution different arbitrary cut-points to define exposure and non-exposure were used for all risk factors. Using different cut-points for exposure and non-exposure yielded similar results for the BMI distributions by risk factors high TV consumption (fig. 3a–d) and for all other risk factors (data not shown) as compared to initial cut-offs and analyses.

## Discussion

Recently two quantitative reviews reported an impact of breastfeeding on upper percentiles represented by childhood obesity [20,21], while the mean difference between breast- and formula fed individuals was low in another quantitative review [28]. This possible contradiction could be due to the observation made in the data set used for this paper that mainly upper quantiles of the BMI distribution are affected by the exposure breast- or formula feeding, respectively. Additionally, we demonstrated that a similar single shift of the upper tail was also found for maternal smoking in pregnancy, frequent TV watching, low meal frequency and poor parental education, whereas the entire BMI distribution with an accentuation on the upper tail was shifted in relation to maternal overweight and high weight gain in the first 2 years of life.

This observation may be relevant with respect to the choice of the outcome variable and corresponding statistical analyses. If the exposure is only associated with a shift of the upper tail of the distribution of a continuous outcome parameter, a true association may be missed if testing refers to the mean by a linear regression model with the continuous outcome parameter as response variable.

Additionally such a shift in the upper part of the distribution only may be an indicator of an effect modification of the exposure confined to a subpopulation of the exposed. The effect modifier may be another environmental exposure or genetic predisposition. Such a gene-environmental exposure interaction was recently demonstrated for passive smoking and asthma in children: a genetically determined deficiency of glutathione S transferase (GST) enzymes involved in the detoxification of environmental tobacco smoke (ETS) accounted for a massive increase in the risk for current asthma and asthma symptoms such as wheeze and shortness of breath [29]. It might well be possible that susceptibility to known risk factors for childhood obesity is confined to children with hitherto unknown genetic polymorphisms.

There are some limitations with regard to the data available for this study and the analysis. The observed effects on parts of the BMI distribution only need to be confirmed in other data sets. Additionally, the corresponding curves were based on univariate analyses: a single shift of the upper tail observed for any of the seven risk factors might be caused by any other of the seven. However, this seems to be unlikely, since these risk factors were proven independent in multivariate logistic regression models with overweight and obesity as outcomes [16,30].

Another important issue is the possibility of underlying dose response effects responsible for skewed shifts of the BMI distributions. It is likely that exposed children differ in dose of exposure. Such a dose variation might explain a non-uniform shift of metric response distributions, in our case BMI, which cannot be assessed by using binary variables for originally continuous exposures. However, choosing different cut-points for binary exposure variables yielded similar results for all exposure categories.

## Conclusion

There are some indicators of health measured on continuous scales, which are commonly converted to categorical outcomes: e.g. BMI to obesity, blood pressure to hypertension. In order to assess the impact of presumed risk factors on these outcomes it may be useful to assess both, the impact on the entire distribution and on parts of the distribution. If only parts of the distribution are shifted by the risk factor considered, this finding may be an indicator of an interaction of the risk factor with other environmental risk factors or with a genetic predisposition. Linear regression models might be difficult in the assessment of risk factors not affecting the entire BMI distribution.

## Competing interests

The author(s) declare that they have no competing interests.

## Authors' contributions

AMT suggested the idea for the article, participated in the statistical analyses and wrote the manuscript. RvK and AB were involved in writing the manuscript and revising it critically for important intellectual content. SR performed the statistical analyses and was involved in writing the manuscript and revising it critically for important intellectual content. All authors approved the final version of the manuscript.

## Pre-publication history

The pre-publication history for this paper can be accessed here:



## References

[B1] Cole TJ, Bellizzi MC, Flegal KM, Dietz WH (2000). Establishing a standard definition for child overweight and obesity worldwide: international survey. Bmj.

[B2] Poskitt EM (1995). Defining childhood obesity: the relative body mass index (BMI). European Childhood Obesity group. Acta Paediatr.

[B3] Zwieauer K, Wabitsch M (1997). Relativer Body Mass index (BMI) zur Beurteilung von Übergewicht und Adipositas im Kindes- und Jugendalter: Empfehlungen der European Childhood Obesity Group. Monatsschr Kinderheilkd.

[B4] Flegal KM, Troiano RP (2000). Changes in the distribution of body mass index of adults and children in the US population. Int J Obes Relat Metab Disord.

[B5] Strauss RS, Pollack HA (2001). Epidemic increase in childhood overweight, 1986-1998. Jama.

[B6] Kalies H, Lenz J, von Kries R (2002). Prevalence of overweight and obesity and trends in body mass index in German pre-school children, 1982-1997. Int J Obes.

[B7] Kautiainen S, Rimpela A, Vikat A, Virtanen SM (2002). Secular trends in overweight and obesity among Finnish adolescents in 1977-1999. Int J Obes Relat Metab Disord.

[B8] Thomsen BL, Ekstrom CT, Sorensen TI (1999). Development of the obesity epidemic in Denmark: cohort, time and age effects among boys born 1930-1975. Int J Obes Relat Metab Disord.

[B9] Toschke AM, Lüdde R, Eisele R, von-Kries R (2005). The obesity epidemic in young men is not confined to low social classes--a time series of 18-year-old German men at medical examination for military service with different educational attainment. Int J Obes (Lond).

[B10] Troiano RP, Flegal KM (1998). Overweight children and adolescents: description, epidemiology, and demographics. Pediatrics Pediatrics.

[B11] Rasmussen F, Johansson M (1998). The relation of weight, length and ponderal index at birth to body mass index and overweight among 18-year-old males in Sweden. Eur J Epidemiol.

[B12] Locard E, Mamelle N, Billette A, Miginiac M, Munoz F, Rey S (1992). Risk factors of obesity in a five year old population. Parental versus environmental factors. Int J Obes Relat Metab Disord.

[B13] Stunkard AJ, Sorensen TI, Hanis C, Teasdale TW, Chakraborty R, Schull WJ, Schulsinger F (1986). An adoption study of human obesity. N Engl J Med.

[B14] Ong KK, Loos RJ (2006). Rapid infancy weight gain and subsequent obesity: systematic reviews and hopeful suggestions. Acta Paediatr.

[B15] Toschke AM, Grote V, Koletzko B, von Kries R (2004). Identifying children at high risk for overweight at school entry by weight gain during the first 2 years. Arch Pediatr Adolesc Med.

[B16] Toschke AM, Küchenhoff H, Koletzko B, von Kries R (2005). Meal frequency and childhood obesity. Obesity Research.

[B17] Dietz WH, Gortmaker SL (1985). Do we fatten our children at the television set? Obesity and television viewing in children and adolescents. Pediatrics Pediatrics.

[B18] Robinson TN (1999). Reducing children's television viewing to prevent obesity: a randomized controlled trial. Jama.

[B19] Toschke AM, Vignerova J, Lhotska L, Osancova K, Koletzko B, von Kries R (2002). Overweight and obesity in 6- to 14-year-old Czech children in 1991: protective effect of breast-feeding. J Pediatr.

[B20] Arenz S, Rückerl R, Koletzko B, von-Kries R (2004). Breast-feeding and childhood obesity--a systematic review. Int J Obes Relat Metab Disord.

[B21] Harder T, Bergmann R, Kallischnigg G, Plagemann A (2005). Duration of breastfeeding and risk of overweight: a meta-analysis. Am J Epidemiol.

[B22] Gillman MW, Rifas-Shiman SL, Camargo CA, Berkey CS, Frazier AL, Rockett HR, Field AE, Colditz GA (2001). Risk of overweight among adolescents who were breastfed as infants. Jama.

[B23] Owen CG, Martin RM, Whincup PH, Smith GD, Cook DG (2005). Effect of infant feeding on the risk of obesity across the life course: a quantitative review of published evidence. Pediatrics Pediatrics.

[B24] Toschke AM, Koletzko B, Slikker W, Hermann M, von Kries R (2002). Childhood obesity is associated with maternal smoking in pregnancy. Eur J Pediatr.

[B25] Toschke AM, Montgomery SM, Pfeiffer U, von Kries R (2003). Early intrauterine exposure to tobacco-inhaled products and obesity. Am J Epidemiol.

[B26] Toschke AM, Martin RM, von KR, Wells J, Smith GD, Ness AR (2007). Infant feeding method and obesity: body mass index and dual-energy X-ray absorptiometry measurements at 9-10 y of age from the Avon Longitudinal Study of Parents and Children (ALSPAC). Am J Clin Nutr.

[B27] Team RDC (2006). R: A Language and Environment for Statistical Computing.

[B28] Owen CG, Martin RM, Whincup PH, vey-Smith G, Gillman MW, Cook DG (2005). The effect of breastfeeding on mean body mass index throughout life: a quantitative review of published and unpublished observational evidence. Am J Clin Nutr.

[B29] Kabesch M, Hoefler C, Carr D, Leupold W, Weiland SK, von ME (2004). Glutathione S transferase deficiency and passive smoking increase childhood asthma. Thorax.

[B30] von Kries R, Toschke AM, Wurmser H, Sauerwald T, Koletzko B (2002). Reduced risk for overweight and obesity in 5- and 6-y-old children by duration of sleep--a cross-sectional study. Int J Obes Relat Metab Disord.

